# Non-Targeted Metabolomics Analysis Revealed the Characteristic Non-Volatile and Volatile Metabolites in the *Rougui* Wuyi Rock Tea (*Camellia sinensis*) from Different Culturing Regions

**DOI:** 10.3390/foods11121694

**Published:** 2022-06-09

**Authors:** Kai Xu, Caiyun Tian, Chengzhe Zhou, Chen Zhu, Jingjing Weng, Yun Sun, Yuling Lin, Zhongxiong Lai, Yuqiong Guo

**Affiliations:** 1 College of Horticulture, Fujian Agriculture and Forestry University, Fuzhou 350002, China; xukai97@foxmail.com (K.X.); cytian1997@foxmail.com (C.T.); chengzhechou@foxmail.com (C.Z.); zhuchen19921118@foxmail.com (C.Z.); w609724642@foxmail.com (J.W.); sunyun1125@126.com (Y.S.); buliang84@163.com (Y.L.); laizx01@163.com (Z.L.); 2 Tea Industry Research Institute, Fujian Agriculture and Forestry University, Fuzhou 350002, China; 3 Institute of Horticultural Biotechnology, Fujian Agriculture and Forestry University, Fuzhou 350002, China

**Keywords:** Rougui Wuyi Rock tea, LC-MS, GC-MS, characteristic components, flavor quality

## Abstract

Rougui Wuyi Rock tea (WRT) with special flavor can be affected by multiple factors that are closely related to the culturing regions of tea plants. The present research adopted non-targeted metabolomics based on liquid chromatography–mass spectrometry (LC-MS) and gas chromatography–mass spectrometry (GC-MS), aroma activity value method (OAV), and chemometrics to analyze the characteristic metabolites of three Rougui WRTs from different culturing regions. The results of sensory evaluation showed that the three Rougui Wuyi Rock teas had significantly different flavor qualities, especially in taste and aroma. Rougui (RG) had a heavy and mellow taste, while cinnamon-like odor Rougui (GPRG) and floral and fruity odor Rougui (HGRG) had a thick, sweet, and fresh taste. The cinnamon-like odor was more obvious and persistent in GPRG than in RG and HGRG. HGRG had floral and fruity characteristics such as clean and lasting, gentle, and heavy, which was more obvious than in RG and GPRG. The results of principal component analysis (PCA) showed that there were significant metabolic differences among the three Rougui WRTs. According to the projection value of variable importance (VIP) of the partial least squares discriminant analysis (PLS–DA), 24 differential non-volatile metabolites were identified. The PLSR analysis results showed that rutin, silibinin, arginine, lysine, dihydrocapsaicin, etc. may be the characteristic non-volatiles that form the different taste outlines of Rougui WRT. A total of 90 volatiles, including aldehydes, alcohols, esters, and hydrocarbons, were identified from the three flavors of Rougui WRT by using GC-MS. Based on OAV values and PLS-DA analysis, a total of 16 characteristic volatiles were identified. The PLSR analysis results showed that 1-penten-3-ol, α-pinene, 2-carene, β-Pinene, dehydrolinalool, adipaldehyde, D-limonene, saffron aldehyde, and 6-methyl-5-hepten-2-one may be the characteristic volatiles that form the different aroma profile of Rougui WRT. These results provide the theoretical basis for understanding the characteristic metabolites that contribute to the distinctive flavors of Rougui WRT.

## 1. Introduction

Oolong tea (*Camellia sinensis*), a typical semi-fermented tea, is well-known for its distinctive flavor and multiple health benefits and is consumed worldwide [1,2,3]. Wuyi Rock tea (WRT), a prestigious and distinctive subcategory of oolong tea produced mainly in the Wuyi mountains in northern Fujian Province, is renowned for its unique ‘rock flavor’ of rich palatability and long-lasting fragrance [4,5]. To date, there are more than 200 cultivars used to process WRT [6]. Among them, Rougui, one of the dominant cultivars of WRT, is planted widely due to its distinctive and stable varietal features [7]. The WRT processed by Rougui cultivars is considered one of the highest-ranking oolong teas, with the intensity of a spicy and cinnamon-like odor and a mellow and heavy taste [8]

Taste and aroma are the pivotal factors in evaluating the quality of tea and are dependent on the secondary metabolites of tea, including the taste compounds and aroma compounds [9,10]. The palatability of tea is mainly attributed to the interactions between taste compounds at the oral physiological levels, which is closely related to specific taste compounds and their content levels [10,11]. Likewise, the odor and fragrance of tea are correlated to the levels of volatile components that contribute to the various aroma characteristics of tea [12]. Taste and aroma are important indicators in judging the quality of WRT, accounting for 35% and 30% of the total sensory evaluation of oolong tea, respectively [13]. A previous study found that tea cultivars and the manufacturing process, together with the special environments of tea plant growth, were the main factors influencing the unique flavor of WRT [14]. Related research about the influence of the manufacturing process and tea cultivars on the content of metabolites that form WRT flavor has been published in several studies [6,15,16]. In addition to the manufacturing process and tea cultivars, many studies have proven that the unique flavor of WRT is determined by the contents of the initial metabolites in the fresh leaves of the tea plant, which hinge on environmental factors [17,18,19]. Wuyi mountain exists in a complex and diverse geographical environment [20]. Previous studies have proved that under the conditions of the same cultivar, tea plant age, production season, and manufacturing process, the metabolite related to the flavor quality of WRT from different microclimate areas varied greatly [20,21,22]. The main differential metabolites included taste metabolites such as amino acids, phenolic acids, flavonol, flavanols, and theaflavin., and volatile metabolites such as alkenes, alkanes, terpenes, and aldehydes. [8]. The earlier studies mainly focused on investigating the characteristic metabolites between authentic rock tea, half rock tea, riverbank tea, and the tea grown outside of Wuyi mountain [21,23,24,25]. However, according to the actual situations of tea plants growth, many tea plants were grown in some peaks formed of protuberant rock (such as Matou Rock, Baoguo Rock, Yingzui Rock, etc.) and pit valleys (such as Huiyuan pit, Daoshui pit, and Niulan pit), where there exist specific microclimates conducive to the growth of tea plants that affects the metabolites of the tea. The special flavor and characteristic metabolites of WRT manufactured with tea leaves from those culturing regions remain largely undetermined. 

Metabolomics is perceived as a kind of comprehensive and efficient omics methodology applied for analyzing total metabolites in organisms [26]; it has been widely plotted for investigating the effects of environment [27,28], tea cultivar [1], and manufacturing process [15,16] on tea metabolites. In this study, non-targeted metabolomics, which was based on liquid chromatography-mass spectrometry (LC-MS) and gas chromatography-mass spectrometry (GC-MS), was used to verify the characteristic non-volatile and volatile metabolites of Rougui WRT. Furthermore, odor activity values (OAV) and multivariate statistical analysis, including principal component analysis (PCA), hierarchical cluster analysis (HCA), partial least squares discrimination analysis (PLS-DA), and partial least squares regression (PLSR), were performed to reveal the different metabolites of WRT. This study will provide insight into ascertaining the characteristic metabolites that contributed to forming the distinctive flavors of Rougui WRT from different culturing regions.

## 2. Materials and Methods

### 2.1. Chemicals

The methanol (≥99.0%) and acetonitrile (≥99.0%) were purchased from Thermo (Shanghai, China). The 2-chlorophenylalanine (98.5%), ammonium formate (≥99.0%), and formic acid (LC-MS grade) were purchased from Aladdin (Shanghai, China), Sigma-Aldrich (Shanghai, China), and TCI (Shanghai, China), respectively. The N-alkane (C7–C40) was purchased from Aoke biology research Co., Ltd. (Beijing, China) for determining the compounds’ retention indices (RIs) in GC analyses. The ethyl caprate (LC-MS grade) was purchased. The water was prepared using a Milli-Q water purification system (Millipore, Billerica, MA, USA).

### 2.2. Tea Samples

Fresh tea leaves of the Rougui cultivar were gathered from different tea-culturing regions, including peaks formed of protuberant rock (Matou Rock), pit valleys (Huiyuan pit), and general tea-culturing regions (Xin Village) of Wuyi mountain in Fujian province (Figure S1) in April 2021. Subsequently, tea leaves were made into Rougui WRT by the professional tea maker of Yong gan Hua Tea Industry, Co., Ltd. (Wuyi Mountain, Wuyishan City, China) using a uniform manufacturing method (withering, fermentation, panning, rolling, drying, and roasting) for Wuyi rock tea according to the geographic location–flavor indications (GB/T 18745-2006). The experiment was completed using three biological replicates. The three types of Rougui WRT, which were made from the tea leaves of Matou Rock, Huiyuan pit, and Xin Village, were called cinnamon-like odor Rougui (GPRG), floral and fruity odor Rougui (HGRG), and Rougui (RG), respectively. All samples were packed in vacuum-sealed bags for LC-MS and GC-MS analysis. 

### 2.3. Sensory Evaluation

Tea samples were evaluated and scored by seven professional and trained sensory recognition panelists (four females and three males, 30 to 50 years old) from the Fujian Agricultural and Forestry University. All panelists had more than five years of descriptive sensory analysis experience with the tea. According to the methodology for the sensory evaluation of tea (GB/T 23776-2018), 110 mL of boiling water was added to 5 g of each tea sample in separate teacups with their lids for 5 min to obtain tea infusion. Then, the intensity values (0–10), taste descriptors (mellow, bitterness, umami, astringency, and thick), and aroma descriptors (floral, fruity, cinnamon-like, and roasted) of each Rougui WRT infusion were subjected to a sensory test by the seven panelists. A scale from 0 to 10 (where 0 was none or no perception and 10 was extremely strong) as described in a previous study [29] was used to symbolize intensity values. 

### 2.4. LC-MS Analysis of Non-Volatile Compounds

The non-volatile compounds in three tea samples were extracted based on LC-MS. The 0.6 mL 2-chlorophenyl alanine (4 ppm) methanol (−20 °C) was added into a 2 mL EP tube containing 200 mg (±1%) of tea samples and smashed using a tissue grinder. After centrifugation (10 min, 12,000 rpm, 4 °C), the supernatant was collected and filtered through a 0.22 µm membrane for LC-MS detection.

The Thermo Ultimate 3000 (Thermo Fisher Scientific, Waltham, MA, USA) equipped with ACQUITY UPLC^®^ HSS T3 column (1.8 µm, 2.1 mm × 150 mm) was used for the non-volatile compounds. The mobile phase, consisting of 5 mM ammonium formate in H_2_O (A), acetonitrile (B), 0.1% formic acid in water (C), and 0.1% formic acid in acetonitrile (D), was used at a flow rate of 0.25 mL/min. The elution gradient program was set as follows: 0~1.0 min, 2% B/D, 1.0~9.0 min, 2~50% B/D; 9.0~12 min, 50~98% B/D; 12.0~13.5 min, 98% B/D; 13.5~14.0 min, 98~2% B/D; 14.0~20 min, 2% D. The temperatures of column and sample manager were set at 40 °C and 8 °C, respectively. 

Mass spectrometry was executed using a Thermo Q Exactive Focus mass spectrometer (Thermo Fisher Scientific, Waltham, MA, USA) equipped with heated electrospray ionization (ESI) probe, and the parameters were set as follows. Electrospray ionization was performed in both positive and negative ionization modes with spray voltages of 3.5 kV and −2.5 kV, respectively. The flow rates of the sheath gas and auxiliary gas were 30 and 10 arbitrary units. The capillary temperature was 325 °C. The resolution of the full-scan MS was set as 70,000, and the mass scan range was mass-to-charge ratio (*m*/*z*) 81–1000. Each sample was analyzed in triplicate.

### 2.5. GC-MS Analysis of Volatile Compounds

A Clarus SQ 8T gas chromatograph-mass spectrometer (Perkin Elmer, New York, NY, USA) equipped with an Elite-5MS column (30.0 m × 0.25 mm × 0.25 μm; Perkin Elmer) was used for the volatile compounds analyses. The GC-MS analytical procedure was conducted according to Wang et al. [30] with minor changes. The temperature was programmed at 50 °C for 5 min, increased at 3 °C/min to 125 °C, and retained for 2 min; then increased at 5 °C/min to 180 °C and held for 3 min; and finally increased at 15 °C/min to 230 °C, and held for 5 min. The flow rate of the carrier gas (helium, 99.999%) was 1 mL/min. The MS spectrometer was operated in electron impact mode with electron energy 70 eV and a scan range of *m*/*z* 45–500. The ion source and mass spectrum transfer-line temperatures were 230 °C and 250 °C, respectively. The volatile peaks were identified by matching the National Institute of Standards and Technology (NIST) mass spectral database and retention index (RI, determined by n-alkane C7–C40). The chemical structures, names, and odors of the volatile constituents were determined according to PubChem (https://pubchem.ncbi.nlm.nih.gov accessed on 8 July 2021) and the Good Scents Company Information System (http://www.thegoodscentscompany.com accessed on 8 July 2021). The relative contents of the volatile constituents (in μg/L) were calculated on the internal standard method [31].

### 2.6. Odor Activity Values (OAVs) Calculation

*OAV* was calculated by dividing the calculated concentration of the volatile compound by its odor threshold in water and was used to evaluate the contributions of the volatile compounds to the aroma of tea samples. It is generally believed that *OAV* ≥ 1 of volatile compounds contributes to the flavor of samples [32]. The calculation formula for *OAV* value is:

$$OAV_{i}\;={\frac{C_{i}}{OT}}_{i}$$

Note: *C_i_* (ug/kg) was the content of the volatile compounds; *OT_i_* (ug/kg) was the aroma threshold of volatile components in water [33].

### 2.7. Statistical Analysis

All data were presented as mean ± standard statistical (SD) of three replicates. The mean and standard deviation of date was calculated by Microsoft Excel 2010. The statistical significance among the different flavors of Rougui tea was determined by one-way ANOVA and Duncan’s multiple range test using SPSS (Version 25.0, Armonk, NY, USA). TBtools (https://github.com/CJ-Chen/TBtools accessed on 8 July 2021) was used for heat-map and hierarchical cluster analysis of the different metabolites and volatile components. The principal component analysis (PCA), hierarchical cluster analysis (HCA), partial least squares discrimination analysis (PLS-DA), and partial least squares regression (PLSR) were performed using SIMCA 14.1 software (Umetrics, Umea, Sweden). 

## 3. Results

### 3.1. Sensory Quality of Rougui Wuyi Rock Tea 

**Figure 1:**
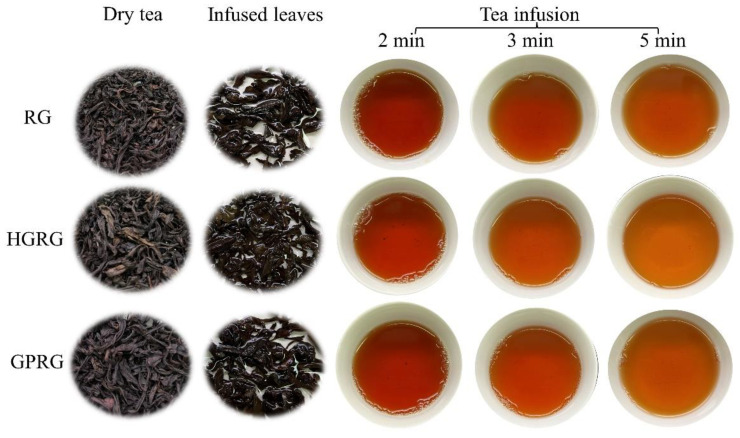
The appearance and infusion colors of Rougui WRT.

The sensory quality of Rougui WRT was investigated. The sensory evaluation findings (Table 1) showed that the appearance was blueish-auburn in all the Rougui teas, with bloom, tightness, and neatness in GPRG and HGRG and tighter, more even RG (Figure 1). An orange-red and bright liquor color was defined in all three Rougui WRTs, and GPRG and HGRG were clear. The cinnamon-like odor existed in RG and GPRG, but it was more obvious and persistent in GPRG. The fruity aroma was evaluated in GPRG and HGRG, and the flowery aroma was noticed in HGRG which was characterized as clean and lasting, gentle, and heavy. In the sensory quality of taste, the mark of all the Rougui WRT was rock flavor and mellow, but the rock flavor was more highlighted in GPRG and HGRG than in RG. RG had a heavy and mellow taste, while GPRG and HGRG had a thick, sweet, and fresh taste. Furthermore, the flowery aroma was discovered in HGRG’s liquor. Infused leaf of the three samples had the characteristics of brightness and evenness, but RG had a softer infused leaf. 

### 3.2. The Non-Volatile Components in Rougui WRT

#### 3.2.1. Non-Volatile Metabolite Profiling of Rougui WRT

**Figure 2:**
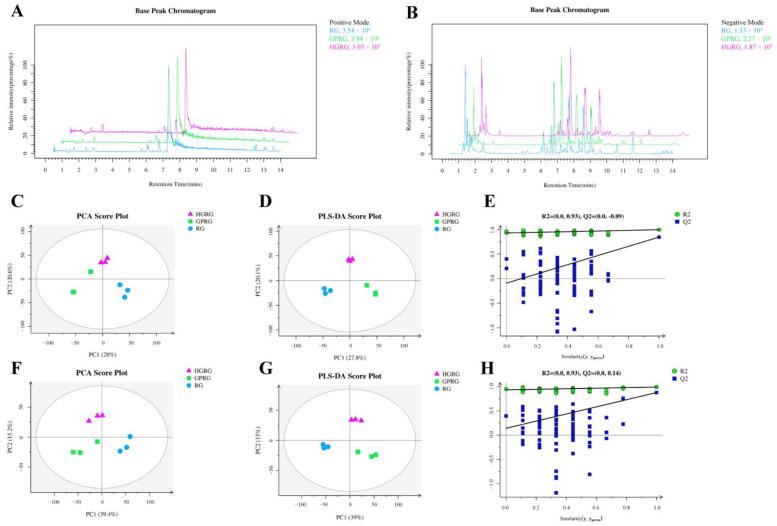
Multivariate statistical analysis results for the differences in the metabolites of the Rougui WRTs. (**A**) LC-MS base peak chromatograms of the extracts of Rougui WRT in positive ionization mode; (**B**) LC-MS base peak chromatograms of the extracts of Rougui WRT in negative ionization mode; (**C**) principal component analysis (PCA)-X scores in positive ion mode; (**D**) partial least squares-discriminant analysis (PLS-DA) score in positive ion mode; (**E**) PLS-DA model validation in positive ion mode; (**F**) PCA-X score in negative ion mode; (**G**) PLS-DA score in negative ion mode; (**H**) PLS-DA model validation in negative ion mode.

To authenticate the kind and difference of metabolites in RG, HGRG, and GPRG, the non-targeted analysis based on LC-MS were plotted to assess non-volatile metabolites. A total of 519 non-volatile metabolites were tested (Table S1). The base peak chromatograms (BPC) of RG, HGRG, and GPRG were performed in positive and negative ion modes and showed that all samples were characterized by strong signal detection by mass spectrometry, large peak capacity, and high separation degree (Figure 2A,B). To explore the differences in the metabolites of the three Rougui WRTs, multivariate data analysis based on all non-volatile metabolites was conducted. A PCA score plot showed a clear separation of the samples of RG, HGRG, and GPRG in positive and negative ion modes (Figure 2C,F), indicating significant differences in the non-volatile metabolites of RG, HGRG, and GPRG. The PLS-DA, which is a supervised analysis method, was employed to investigate the differences between the three Rougui WRTs (Figure 2D,G). According to the PLS-DA score plot, the non-volatile components of the three Rougui WRTs had obvious differences, similar to PCA results. The permutation plots of PLS-DA showed that the model had an effectively predictive ability and without overfitting (Figure 2E,H).

#### 3.2.2. Identification and Analysis of Differential Metabolites of Rougui WRT

**Figure 3:**
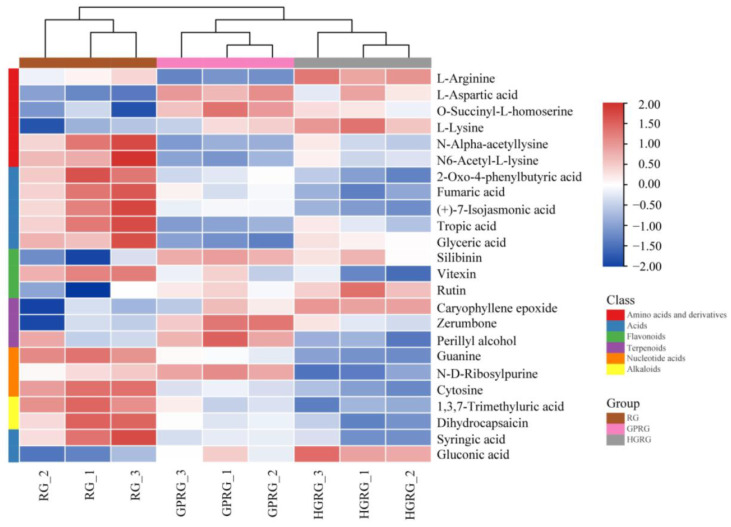
A hierarchical clustering heatmap of 24 different non-volatile metabolites of Rougui WRT.

The variable importance in the project (VIP) > 1 and *p* < 0.05 were interpreted as different metabolites. A total of 24 different metabolites were identified, including six amino acids and their derivatives, seven organic acids, three flavonoids, three terpenoids, three nucleotide acids, and two alkaloids (Table S2). Hierarchical clustering based on heat map visualization was used to investigate the differences in the contents of 24 metabolites in three samples (Figure 3). The result showed that the content of L-arginine, L-aspartic acid, L-lysine, silibinin, caryophyllene epoxide, and rutin was higher in HGRG and GPRG than that in RG, while the RG contained higher levels of four organic acids (fumaric acid, tropic acid, glyceric acid, and syringic acid), two nucleotide acids (guanine and cytosine), and one alkaloid (dihydrocapsaicin) compared with HGRG and GPRG. Additionally, the levels of zerumbone and perillyl alcohol were significantly higher in GPRG than in RG and HGRG. These results suggest those different metabolites as the important compounds for identifying Rougui WRT with different flavors in different tea-culturing regions.

#### 3.2.3. Correlation Analysis between Taste Profiles and Characteristic Non-Volatile Metabolites of Rougui WRT

**Figure 4:**
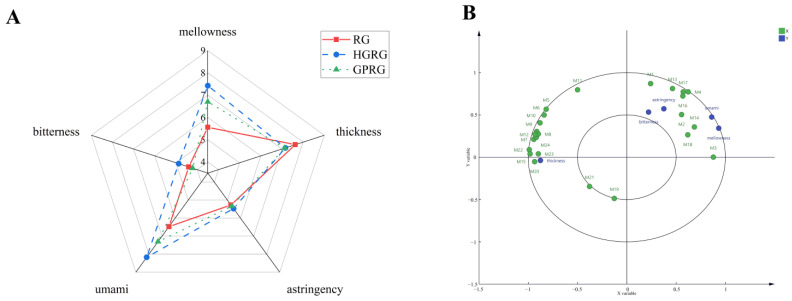
(**A**) Spider plots of the taste profiles of three Rougui WRTs; (**B**) PLSR analysis between taste properties and characteristic non-volatile metabolites (VIP > 1 and *p* < 0.05) of the three Rougui WRTs. X-axi. the 24 non-volatile metabolites (VIP > 1 and *p* < 0.05); Y. the taste properties.

To understand the sensory properties of Rougui WRT, the taste properties of the three Rougui WRTs, they were evaluated by a sensory panel (Figure 4A). Higher mellowness and umami were found in HGRG, followed by GPRG, and finally by RG, whereas RG had the stronger thickness than that in HGRG and GPRG. Additionally, moderate-intensity bitter and astringent tastes both existed in the three Rougui WRTs.

To further understand and ascertain the characteristic non-volatile metabolites contributing to the taste properties of the three WRTs, the PLSR model with a single sensory attribute (Y) and 24 differential non-volatile metabolites (X) was performed (Figure 4B). The O-succinyl-L-homoserine (M3), L-aspartic acid (M2), etc. correlated to mellowness and umami, whereas L-arginine (M1), L-lysine (M4), rutin (M16), silibinin (M14), and the other non-volatile metabolites correlated well with bitterness and astringency. Additionally, a number of non-volatile metabolites such as vitexin (M15), dihydrocapsaicin (M24), and many organic acids made a positive contribution to the thickness of Rougui WRT.

### 3.3. Volatile Components in Rougui WRT

#### 3.3.1. Composition of Volatile Components of Rougui WRT

**Figure 5:**
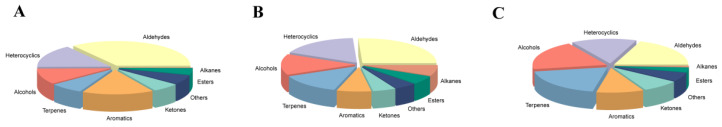
The ratios of the 90 volatile components in Rougui WRT. (**A**) The ratios of the different types of RG. (**B**) The ratios of the different types of HGRG. (**C**) The ratio of different types of GPRG.

A total of 90 volatile components, including 9 alcohols, 14 aldehydes, 13 esters, 19 terpenes, 7 aromatics, 7 alkenes, 5 ketones, 10 heterocyclics, and 6 others, were authenticated by GC-MS in Rougui WRT (Table S3). Among the 90 volatile components, 81 were detected in HGRG and GPRG, whereas only 61 were measured in RG, suggesting that the numbers of volatile components in RG, HGRG, and GPRG might be one of the reasons for the differences in the aromas of the three Rougui WRTs. To further investigate the volatile components in the three Rougui WRTs, we analyzed the proportions of the 90 volatile components in RG, GPRG, and HGRG (Figure 5). The aldehydes took up a dominant portion in both RG and HGRG, which had the highest proportion (35.05% and 25.89%, respectively). In addition to aldehydes, the aromatics (15.67%) and alcohols (10.37%) were the most abundant in RG, whereas the alcohols (13.61%) and terpenes (13.42%) were the most abundant in HGRG. The GPRG had a lower proportion of aldehydes (19.25%) and higher proportions of alcohols (19.24%) and terpenes (19.00%) than RG and HGRG. 

To explore the differences in volatile components of RG, HGRG, and GPRG, we calculated the relative content of 90 volatile components (Table 2). The pentanal had the highest content in RG (1327.27 μg/kg), followed by p-xylene, toluene, N-ethylpyrrole, and 1-penten-3-ol, followed by 2-methylbutyraldehyde, which had the highest content in both HGRG and GPRG. In addition to 2-methylbutyraldehyde, N-ethylpyrrole, 1-penten-3-ol, 2-ethylfuran, hexadecane, and 3-methylbutyraldehyde were the most abundant in HGRG, whereas dehydrolinalool, D-limonene, 1-penten-3-ol, (Z)-α-α-5-trimethyl-5-vinyltetrahydrofuran-2-methanol, β-pinene, and toluene, components with a strong floral, fruity, citrus-like, sweet, and woody odor, were abundant in GPRG. This indicated that discrepancies in the proportions and contents of the volatile components may cause the different aroma profiles of the three Rougui WRTs. 

#### 3.3.2. Analysis of Characteristic Volatile Components of Rougui WRT

The contributions of volatile components to tea aroma cannot completely depend on the amount of each volatile, so we used OAV (≥1) to calculate the significance for the characteristic volatile components in Rougui WRT (Table S4). The result showed that a total of 24 volatile components revealed OAVs ≥1 in RG, HGRG, and GPRG, including 4 alcohols, 8 aldehydes, 2 esters, 8 terpenes, 1 aromatic, and 1 ketone, which could be interpreted as characteristic volatile components of the aroma profile of Rougui WRT. Among them, linalool, 2-methylbutyraldehyde, adipaldehyde, phenylacetaldehyde, β-cyclocitral, (Z)-caproic acid 3-hexenyl ester, hexanoic acid hexyl ester, D-limonene, α-farnesene, β-pinene, toluene, and 6-methyl-5-hepten-2-one were the volatiles with OAVs ≥1 in all samples. Additionally, pentanal with fruity and roasted, saffron aldehyde with woody-like and saffron-like odor, and 1-octen-3-ol with citrus-like odor were the only volatiles with OAVs above 1 and existed in RG, HGRG, and GPRG alone, respectively.

**Figure 6:**
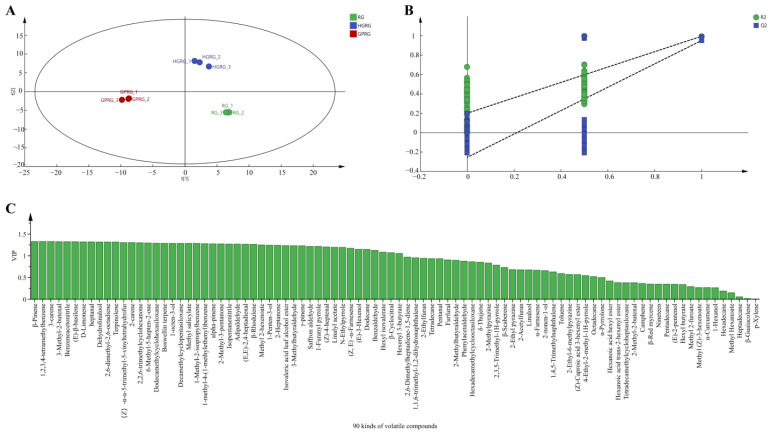
(**A**) The PLS-DA scores for the 90 volatile components of Rougui WRT; (**B**) permutation test plots of the 90 volatile components of Rougui WRT; (**C**) the variable importance in the project (VIP) of the 90 volatile components of Rougui WRT.

To distinguish characteristic volatile components in RG, HGRG, and GPRG, the PLS-DA model (Figure 6) was used to reveal the differences in volatiles among the three Rougui WRTs. According to the PLS-DA score plot (Figure 6A), RG, HGRG, and GPRG were clustered within IV, I, and III quadrants, respectively, suggesting that there were significant separation and differences among the three samples. Furthermore, we found that R^2^Y and Q^2^ were 0.944 and 0.983, respectively, and the intercept of Q^2^ and *Y*-axis was less than 0, which could be interpreted as reflecting that the model had effective predictive ability without overfitting (Figure 6B). Subsequently, the variable importance in the project (VIP) was calculated (Figure 6C), and the volatiles with both VIP >1 and OAVs ≥1 were selected as potential difference volatiles for further identification using relevant published data in the literature and in databases (Table S5). A total of 16 components were identified as characteristic volatile components in RG, HGRG, and GPRG, including 1-penten-3-ol, 1-octen-3-ol, dehydrolinalool, 3-methylbutyraldehyde, adipaldehyde, (Z)-4-heptenal, saffron aldehyde, β-cyclocitral, 3-carene, 2-carene, α-pinene, D-limonene, β-Rhodulene, (E)-β-basilene, β-pinene, and 6-methyl-5-hepten-2-one (Table S5).

#### 3.3.3. Correlation Analysis between Aroma Profiles and Characteristic Volatile Metabolites of Rougui WRT

**Figure 7:**
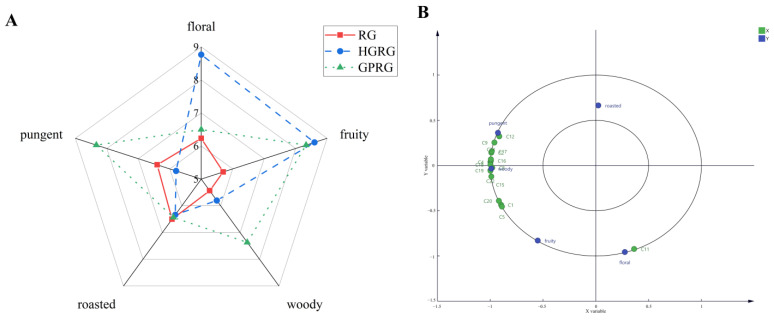
(**A**) Spider plots for the aroma profiles of three Rougui WRT; (**B**) PLSR analysis between the aroma properties and the characteristic volatile metabolites (OAVs ≥ 1 and VIP > 1) of three Rougui WRT. X. 16 volatile metabolites (OAVs ≥ 1 and VIP > 1); Y. aroma properties.

To explore the aroma profiles of HGRG, GPRG, and RG, their aroma properties (including floral, fruity, woody, pungent, and roasted) were evaluated with a sensory panel (Figure 7A). HGRG had the highest intense floral and fruity odors, whereas GPRG had the strongest pungent and woody odors. Meanwhile, a high fruity odor and moderate pungent and woody odors also existed in GPRG and HGRG, respectively. RG had moderate fruity, floral, woody, and pungent odors. Notable, the pungent odor in RG was stronger than that in HGRG, and a moderate roasted odor existed in all the tea samples.

To further confirm the relationships between the volatiles and the aroma properties, PLSR analysis was applied to correlate the key volatiles (OAVs ≥ 1 and VIP > 1) with the aroma properties (floral, fruity, woody, pungent, and roasted) in the three Rougui WRTs (Figure 7B). Most of the volatiles, including 3-carene (C15, with a citrus-like and woody odor), α-pinene (C17, with woody and herbal odors), β-pinene (C22, woody odor), 6-methyl-5-hepten-2-one (C24, with fruity, herbaceous, pungent, and lemon-like odors), D-limonene (C18, citrus-like and herbal odors) had a strong correlate with the woody and pungent odors. The saffron aldehyde (C11, saffron odor) had a high correlation with a floral aroma, whereas the 1-penten-3-ol (C1, fruity and vegetable-like odors) and 3-methylbutyraldehyde (C5, with peach fatty odor) contributed significantly. 

## 4. Discussion

### 4.1. Rutin, Silibinin, Arginine, Lysine, Dihydrocapsaicin, etc. May Be the Characteristic Non-Volatiles That form the Different Taste Outline of Rougui WRT

Taste is one of the primary factors in evaluating tea quality, which is determined by the types and contents of chemical components [10,34]. The polyphenols, amino acids, alkaloids, carbohydrates, and other components in tea can directly or indirectly affect the bitterness, astringent, umami, and sweetness of tea infusion [35]. Most notably, those components that contributed to the tea flavors were significantly affected by the geographic environment [21]. In this study, we analyzed the non-volatile metabolites of Rougui WRT in different culturing regions based on LC-MS and found that there were significant differences in the compositions of the metabolites in the three Rougui WRT (Figure 2C,F), indicating that the differences in the potential metabolites of Rougui WRT might be strongly influenced by terrain environment. 

According to the results for sensory evaluation, the three Rougui WRTs had different flavors. The formation of tea’s different flavors is closely related to the components and contents of various metabolites [10]. To affirm the characteristic non-volatile metabolites, contributing to the different flavors of Rougui WRT, 24 differential metabolites (VIP > 1 and *p* < 0.05) were identified in the three Rougui WRTs (Table S2). We found that the differential metabolite contents of amino acids and their derivatives and flavonoids in HGRG and GPRG were higher than those in RG, whereas the contents of the organic acids and alkaloids were lower than those in RG (Figure 3). The amino acids and flavonoids, which are the key taste metabolites in the tea, play a crucial role in the umami and thickness of tea infusions, respectively [36,37]. Meanwhile, organic acids also inhibit the bitterness in tea infusions [38]. Thus, it was speculated that the differences in the tastes of the three Rougui WRTs may be due to the difference in those non-volatile metabolites. Subsequently, to further ascertain the specific contributions of non-volatile metabolites on taste properties in the three Rougui WRTs, the PLSR model based on the sensory attributes and the 24 non-volatile metabolites was used to perform correlation analysis. The PLSR analysis result (Figure 4B) showed that many of the metabolites such as vitexin, dihydrocapsaicin, and some organic acids had strong correlations with the thickness. Vitexin and dihydrocapsaicin, which belong to flavonoids and alkaloids, respectively, contributed to the thicknesses of the tea infusions. In this study, RG had higher contents of vitexin, dihydrocapsaicin, and organic acids, suggesting that this may account for the thicker taste of RG compared with HGRG and GPRG. Additionally, some amino acids such as L-aspartic acid and O-succinyl-L-homoserine were associated with umami and mellowness, which may be the pivotal metabolites responsible for the higher umami and mellowness of HGRG and GPRG compared with RG. In addition to contributing to the freshness of tea infusions, some amino acids such as L-arginine and L-lysine are also conducive to bitterness for enhancing the mellowness and thickness of tea taste [39]. In this study, L-arginine, L-lysine, rutin, and silibinin, the non-volatile metabolites had a strong correlation with bitterness and astringency. The palatability of tea is mainly attributed to the interactions between taste compounds at the oral physiological level. It has been proven that rutin has no obvious taste but has the function of enhancing the bitterness of caffeine in tea [40,41]. Some organic acids may be the key metabolites that inhibit the effect of bitterness metabolites such as rutin, L-lysine, and L-arginine. It is conjectured that vitexin and dihydrocapsaicin combined with the effects of many organic acids may be conducive to the heavy taste of RG. 

Interestingly, we also found that terpenoid metabolites, including caryophyllene epoxide, zerumbone, and perillyl alcohol existed in HGRG and GPRG (Table S2 and Figure 3). Caryophyllene epoxide, which is characterized by a floral and fruity aroma [42], was high in HGRG (Figure 3), indicating that caryophyllene epoxide may indirectly enhance the floral and fruity aroma of HGRG. Zerumbone, which is a kind of sesquiterpene, is instrumental in the special spicy aroma of *Syringa pinnatifolia* [43], whereas perillyl alcohol is one of the crucial substances that constitute the outline of the spicy aroma of *Perilla frutescens* [44]. In this study, zerumbone and perillyl alcohol were high in GPRG (Figure 3), suggesting that these metabolites indirectly contribute to the formation of the spicy characteristic of GPRG.

### 4.2. 1-Penten-3-ol, α-Pinene, 2-Carene, β-Pinene, Dehydrolinalool, Adipaldehyde, D-Limonene, Saffron Aldehyde, and 6-Methyl-5-hepten-2-one, etc. May Be the Characteristic Volatiles That Forms the Different Aroma Profile of Rougui WRT

The aroma is another key factor in evaluating tea quality, which is accounted for 30% of the total sensory evaluation of oolong tea [13]. The types and contents of volatile compounds had a crucial effect on forming the aroma characteristics of oolong tea and were greatly affected by the geographical environment. The previous study found that the aroma substances of WRT in different regions had significant differences, including esters, alcohols, aldehydes, terpenes, alkenes, and ketones [8]. In this study, a total of 90 volatiles were determined (including alcohols, aldehydes, esters, terpenes, alkenes, and ketones) in Rougui WRT in different tea-culturing regions using GC-MS. We found that there was a difference in content and proportion of the above volatiles in Rougui WRT from different tea-culturing regions (Table 2 and Figure 5), which was consistent with the result of Xu et al. [24].

Additionally, the characteristic aroma of tea not only depends on the type and content of the volatiles but also is significantly related to the aroma activity values and their synergistic effects [45]. Thus, the OVA was used to evaluate the contributions of the volatile components to the characteristic aromas of Rougui WRT. Then, 16 volatiles with OAVs ≥ 1 and VIP > 1 (Table S5) were screened, suggesting that the above volatiles may be the basis for the distinctive aromas of Rougui WRT in different culturing regions. To prove this hypothesis, PLSR analysis was used to explore the contribution of aroma-active compounds to the aroma properties of three Rougui WRT (Figure 7B). The dehydrolinalool (with green and fruit odors), β-pinene (with woody odor), D-limonene (with citrus-like and herbal odor), 6-methyl-5-hepten-2-one (with fruity, herbaceous, pungent, and lemon-like odors), etc. had strong correlations with the woody and pungent odors. Among the above volatiles, dehydrolinalool is perceived as the main aroma component of Rougui WRT [46]. Interestingly, the dehydrolinalool has also been found to play an important role in forming the spicy aroma of Zijuan tea and green tea made from the tea leaves of ancient tea plants [47,48]. Limonene (with citrus-like and herbal odor) and β-Pinene (woody odor) are the key volatiles in Rougui WRT [46,49], and the limonene has a significant contribution to the spicy odor of *Zanthoxylum bungeanum Maxim* [50]. It is speculated that the aroma profile of HGRG with strong cinnamon-like and woody odors (Table 1 and Figure 7A) may be closely related to the above characteristic volatiles. The 1-penten-3-ol and 3-methylbutyraldehyde correlated with a fruity aroma and had high OAV in HGRG and GPRG, suggesting they would be the characteristic volatiles that contribute to a strong fruity aroma in HGRG and GPRG. Notably, the saffron aldehyde is crucial to forming a floral and fruity aroma of Rougui WRT [49,51]. In this study, saffron aldehyde was highly correlated with the floral aroma (Figure 7B) and was only detected in HGRG, suggesting that saffron aldehyde would be the characteristic volatile of HGRG with the strongest floral aroma compared with RG and GPRG. 

## 5. Conclusions

In this study, LC-MS, GC-MS, and OAV analyses are combined with multivariate statistical analysis to measure the different metabolites of Rougui Wuyi Rock tea. The results showed that amino acids, flavonoids, organic acids, and terpenes are major different metabolites in the three flavors of Rougui teas. The PLSR analysis results showed that rutin, silibinin, arginine, lysine, and dihydrocapsaicin may be used to identify Rougui WRTs of diverse flavors. A total of 90 volatile components were detected with GC-MS, including alcohols, aldehydes, esters, terpenes, aromatics, alkenes, ketones, heterocyclics, and others. In addition, the stoichiometric method, OAV analysis, and PLSR analysis were used to screen the characteristic metabolites, including dehydrolinalool, 3-carene, limonene, 6-methyl-5-hepten-2-one. The result indicated that dehydrolinalool, 2-carene, limonene, 6-methyl-5-hepten-2-one, and saffron aldehyde were the characteristic aroma components that formed the different aroma profiles of Rougui WRT. However, the major limitation of this study is that the tea samples are small and OAV also has certain limitations in the analysis of characteristic flavor compounds. Therefore, there is a need to expand tea samples and conduct aroma reorganization and reduction experiments to comprehensively determine the characteristic aroma compounds. 

## Figures and Tables

**Table 1:** Sensory evaluation of Rougui WRT.

Experiment Samples	Appearance	Liquor Color	Aroma	Taste	Infused Leaf
RG	greenish-brown, tighter, and comparatively uniform	orange-red, bright	cinnamon-like aroma normally	strong, mellow, and rocky flavor normally	comparatively bright and uniform
GPRG	greenish-brown and glossy, tighter, and clean uniformity	orange-red, translucent	significant cinnamon-like and lasting aroma, a little fruity	mellow, thick, and umami; significant rocky flavor	bright and uniform
HGRG	greenish-brown and glossy, tighter, and clean uniformity	orange-red, translucent	significant fruity and flowery aroma, long-lasting	mellow, thick, and umami; significant rocky flavor, fusion with flowery	comparatively bright and uniform

**Table 2:** The qualitative results for the volatile components in Rougui WRT.

No.	Compounds	Odour Description	Concentration (μg/kg Dry Weight of Tea Leaves)		
			RG	HGRG	GPRG
	**Alcohols**				
1	1-Penten-3-ol	Fruity, vegetable-like	339.28 ± 23.80 ^c^	604.42 ± 2.63 ^b^	797.70 ± 7.64 ^a^
2	trans-2-pentenol	Mushroom-like	n.d.	73.59 ± 5.30 ^a^	n.d.
3	trans-3-Hexanol	-	38.12 ± 0.41 ^b^	39.23 ± 6.81 ^b^	52.78 ± 3.42 ^a^
4	1-Hexanol	Yeast aroma, Green, cut grass	36.61 ± 0.62 ^a^	24.62 ± 2.67 ^c^	31.07 ± 0.78 ^b^
5	1-octen-3-ol	Waxy, fatty and citrus-, mushroom-like	n.d.	n.d.	6.88 ± 0.18 ^a^
6	cis-α-α-5-trimethyl-5-vinyltetrahydrofuran-2-methanol	-	87.60 ± 6.69 ^c^	124.43 ± 14.90 ^b^	630.06 ± 10.13 ^a^
7	Linalool	Floral, fruity	29.14 ± 6.58 ^c^	88.61 ± 8.97 ^a^	74.41 ± 3.77 ^b^
8	Dehydrolinalool	Floral, fruity	35.52 ± 0.01 ^c^	155.25 ± 19.69 ^b^	1038.99 ± 6.62 ^a^
9	2-nonen-1-ol	-	191.20 ± 0.73 ^a^	23.08 ± 4.15 ^c^	67.72 ± 2.87 ^b^
	**Aldehydes**				
10	3-Methylbutyraldehyde	Peach Fatty	n.d.	235.45 ± 8.81 ^b^	391.44 ± 13.68 ^a^
11	2-Methylbutyraldehyde	Grassy green	336.00 ± 22.07 ^c^	1327.27 ± 6.16 ^a^	1283.00 ± 14.12 ^b^
12	Pentanal	Fruity, roast	1407.76 ± 7.63 ^a^	n.d.	n.d.
13	2-Methyl-2-butenal	Green, fruity	176.96 ± 5.99 ^a^	193.08 ± 13.66 ^a^	187.85 ± 6.91 ^a^
14	3-Methyl-2-butenal	Sweet, fruity, nutty	43.10 ± 3.23 ^c^	89.89 ± 4.40 ^b^	292.02 ± 12.72 ^a^
15	Adipaldehyde	Green, fruit	124.50 ± 2.45 ^b^	128.49 ± 6.83 ^b^	172.56 ± 9.21 ^a^
16	Furfural	Bread-like, roast	62.34 ± 1.03 ^b^	31.08 ± 8.60 ^c^	91.36 ± 6.66 ^a^
17	cis-4-heptenal	Vegetable-like, roasted	10.40 ± 0.08 ^b^	7.76 ± 2.24 ^c^	23.48 ± 0.22 ^a^
18	heptanal	Fruity, green	10.50 ± 0.03 ^c^	12.70 ± 0.91 ^b^	24.24 ± 0.56 ^a^
19	Benzaldehyde	Sweet, fruity	95.52 ± 4.85 ^b^	83.75 ± 13.74 ^b^	134.30 ± 2.10 ^a^
20	(E,E)-2,4-heptadienal	Green, fruity	8.49 ± 0.02 ^b^	6.58 ± 0.48 ^c^	37.70 ± 0.12 ^a^
21	Phenylacetaldehyde	Grassy green, floral	256.70 ± 1.70 ^a^	7.31 ± 1.43 ^c^	23.28 ± 0.57 ^b^
22	Saffron aldehyde	Woody, saffron	n.d.	10.27 ± 1.39 ^a^	n.d.
23	β-Cyclocitral	Rose-like, herbal smell	27.55 ± 0.73 ^b^	22.10 ± 4.61 ^b^	39.59 ± 1.40 ^a^
	**Esters**				
24	Methyl Hexanoate	Pineapple-like	28.83 ± 0.35 ^a^	22.76 ± 1.26 ^b^	27.88 ± 0.63 ^a^
25	Methyl (Z)-3-hexenoate	Fruity	56.09 ± 0.91 ^a^	61.10 ± 4.16 ^a^	58.76 ± 0.07 ^a^
26	Methyl 2-hexenoate	Pineapple-like	32.17 ± 0.58 ^b^	29.27 ± 4.36 ^b^	68.37 ± 3.48 ^a^
27	Methyl 2-furoate	Fruity, sweet	16.57 ± 0.03 ^a^	6.32 ± 0.34 ^c^	11.70 ± 1.33 ^b^
28	Hexenyl 3-butyrate	-	9.08 ± 0.13 ^b^	13.50 ± 1.09 ^a^	14.47 ± 0.39 ^a^
29	Methyl salicylate	Green, peppermint	n.d.	n.d.	23.39 ± 0.80 ^a^
30	Hexyl butyrate	Apple-like	n.d.	20.18 ± 4.64 ^a^	n.d.
31	Isovaleric acid leaf alcohol ester	Fruity	13.45 ± 0.37 ^c^	20.92 ± 4.68 ^b^	29.92 ± 0.89 ^a^
32	Hexyl isovalerate	Fruity	22.20 ± 0.25 ^b^	30.59 ± 5.11 ^a^	34.88 ± 1.12 ^a^
33	Linalyl acetate	Floral and fruity	n.d.	22.05 ± 5.75 ^a^	n.d.
34	(Z)-Caproic acid 3-hexenyl ester	Green, fruity	63.51 ± 0.99 ^b^	111.09 ± 15.63 ^a^	96.56 ± 3.04 ^a^
35	Hexanoic acid hexyl ester	Fruity	56.08 ± 0.61 ^b^	94.83 ± 12.14 ^a^	52.54 ± 5.48 ^b^
36	Hexanoic acid trans-2-hexenyl ester	Herbal smell	14.60 ± 1.44 ^b^	31.27 ± 5.25 ^a^	13.73 ± 0.97 ^b^
	**Terpenes**				
37	2,6-Dimethylheptadiene-1,5-diene	-	42.73 ± 0.28 ^b^	21.46 ± 3.88 ^c^	68.46 ± 8.33 ^a^
38	Camphene	Camphor-like	n.d.	n.d.	24.54 ± 1.98 ^a^
39	2,6-dimethyl-2,6-octadiene	-	51.34 ± 0.33 ^c^	80.66 ± 11.15 ^b^	281.30 ± 2.81 ^a^
40	3-carene	Citrus-like, woody	n.d.	55.48 ± 3.59 ^b^	198.02 ± 11.50 ^a^
41	2-carene	Grassy green	27.67 ± 0.79 ^b^	37.42 ± 6.66 ^b^	134.28 ± 8.45 ^a^
42	alpha-pinene	Woody, herbal smell	29.39 ± 0.76 ^b^	26.30 ± 3.34 ^b^	130.95 ± 5.89 ^a^
43	D-Limonene	Citrus-like, herbal smell	158.58 ± 0.88 ^c^	245.85 ± 7.03 ^b^	788.46 ± 6.15 ^a^
44	trans-β-basilene	Grassy green, floral	n.d.	41.69 ± 8.16 ^b^	129.95 ± 4.29 ^a^
45	β-Rhodulene	Grassy green, floral	18.75 ± 0.13 ^c^	204.54 ± 13.09 ^b^	362.54 ± 6.32 ^a^
46	gamma-pinene	Woody, herbal smell	46.23 ± 2.41 ^b^	36.49 ± 4.66 ^b^	116.47 ± 8.52 ^a^
47	Terpinolene	Citrus-like, woody	27.67 ± 0.98 ^c^	44.79 ± 6.34 ^b^	154.35 ± 5.43 ^a^
48	β-Scaberene	Spicy, woody	24.39 ± 0.06 ^b^	44.54 ± 6.19 ^a^	41.20 ± 2.03 ^a^
49	α-Curcumene	-	8.82 ± 0.05 ^b^	69.80 ± 7.36 ^a^	12.81 ± 0.16 ^b^
50	(Z, E)-α-Farnesene	-	n.d.	8.05 ± 1.30 ^a^	n.d.
51	α-Farnesene	Citrus-like, herbal smell	26.43 ± 0.21 ^b^	43.75 ± 6.00 ^a^	19.44 ± 1.35 ^b^
52	β-Red myrcene	-	15.11 ± 0.89 ^c^	27.86 ± 2.05 ^a^	21.70 ± 0.64 ^b^
53	δ-Thujene	Woody	n.d.	n.d.	4.60±0.09 ^a^
54	β-Guaiacolene	-	51.41 ± 1.30 ^c^	129.05 ± 14.94 ^a^	71.32 ± 4.77 ^b^
55	Boswellin terpene	-	n.d.	n.d.	111.53 ± 2.82 ^a^
56	β-Pinene	-	162.02±0.51 ^c^	253.34±12.31 ^b^	599.07±1.43 ^a^
	**Aromatic compounds**				
57	Toluene	Sweet	501.97 ± 22.76 ^b^	288.26 ± 12.29 ^c^	558.10 ± 10.72 ^a^
58	p-Xylene	-	505.72 ± 28.42 ^a^	190.99 ± 8.32 ^c^	421.49 ± 6.48 ^b^
59	1-Methyl-2-isopropylbenzene	-	63.31 ± 2.03 ^b^	64.15 ± 10.36 ^b^	322.71 ± 11.59 ^a^
60	1-methyl-4- (1-methylethenyl) benzene	Spicy, clove-like	23.79 ± 0.13 ^b^	26.58 ± 4.61 ^b^	74.00 ± 7.91 ^a^
61	1,2,3,4-tetramethylbenzene	-	n.d.	3.45 ± 0.52 ^b^	11.43 ± 0.08 ^a^
62	1,1,6-trimethyl-1,2-dihydronaphthalene	Licorice-like	36.51 ± 2.18 ^b^	28.24 ± 4.65 ^b^	49.50 ± 7.09 ^a^
63	1,4,5-Trimethylnaphthalene	-	12.71 ± 0.33 ^b^	24.33 ± 3.54 ^a^	8.79 ± 0.55 ^b^
	**Alkanes**				
64	Dodecane	-	n.d.	12.86 ± 3.90 ^b^	18.74 ± 0.14 ^a^
65	Tetradecane	Fruity	11.00 ± 1.21 ^b^	21.35 ± 11.24 ^ab^	27.01 ± 1.81 ^a^
66	Pentadecane	-	n.d.	124.09 ± 1.51 ^a^	n.d.
67	Hexadecane	Orchid-like	n.d.	239.16 ± 7.11 ^a^	31.08 ± 1.39 ^b^
68	Heptadecane	-	n.d.	141.14 ± 6.32 ^a^	32.31 ± 0.35 ^a^
69	Octadecane	-	n.d.	24.02 ± 11.39 ^a^	16.41 ± 0.25 ^a^
70	Nineteen	-	n.d.	17.93 ± 0.17 ^a^	n.d.
	**Ketones**				
71	2-Methyl-3-pentanone	Peppermint-like	217.71 ± 5.83 ^b^	199.01 ± 11.50 ^c^	691.57 ± 5.71 ^a^
72	2-Heptanone	Fruity and sweet	77.83 ± 2.87 ^b^	69.91 ± 5.95 ^b^	143.27 ± 0.69 ^a^
73	6-Methyl-5-hepten-2-one	Fruity, lemon-like	114.74 ± 0.43 ^b^	120.71 ± 9.16 ^b^	254.84 ± 4.53 ^a^
74	2,2,6-trimethylcyclohexanone	Spicy, sweet citrus	22.08 ± 0.40 ^b^	27.31 ± 3.01 ^b^	47.41 ± 3.88 ^a^
75	α-Pyrrolone	Sweet, fruity	n.d.	5.28 ± 0.38 ^a^	3.25 ± 0.19 ^b^
	**Heterocyclic compounds**				
76	2-Ethylfuran	Caramel-like	217.46 ± 4.88 ^b^	372.35 ± 3.95 ^a^	375.61 ± 15.27 ^a^
77	2-Acetylfuran	Sweet, roasted	13.78 ± 0.44 ^a^	10.69 ± 2.55 ^b^	15.60 ± 0.40 ^a^
78	N-Ethylpyrrole	Roasted	397.14 ± 12.58 ^c^	647.99 ± 16.94 ^b^	775.20 ± 6.67 ^a^
79	2,3,5-Trimethyl-1H-pyrrole	-	21.59 ± 0.39 ^b^	44.77 ± 6.31 ^a^	41.86 ± 0.08 ^a^
80	4-Ethyl-2-methyl-1H-pyrrole	-	9.05 ± 0.80 ^b^	21.30±5.93 ^a^	17.69 ± 0.58 ^a^
81	1-Furanyl pyrrole	Fruity	46.57 ± 0.18 ^b^	33.76 ± 7.98 ^c^	116.18 ± 5.05 ^a^
82	2-Methylpyrazine	Nutty, roasted	38.96 ± 1.95 ^ab^	28.47 ± 8.74 ^b^	50.45 ± 6.94 ^a^
83	2-Ethyl pyrazine	Nutty, roasted	15.02 ± 0.58 ^b^	19.63 ± 0.15 ^a^	13.19 ± 0.72 ^c^
84	2-Ethyl-6-methylpyrazine	Roasted potato	186.97 ± 240.61 ^a^	n.d.	n.d.
	**Others**				
85	Decamethylcyclopentasiloxane	-	n.d.	n.d.	222.11 ± 5.86 ^a^
86	Dodecamethylcyclohexasiloxane	-	22.47 ± 0.14 ^b^	132.79 ± 8.68 ^b^	251.62 ± 4.28 ^a^
87	Tetradecamethylcycloheptasiloxane	-	n.d.	n.d.	94.07 ± 3.44 ^a^
88	Hexadecamethylcyclooctasiloxane	-	201.11 ± 4.52 ^a^	25.68 ± 4.33 ^c^	39.50 ± 0.54 ^b^
89	Isopentanenitrile	-	44.11 ± 4.07 ^c^	80.47 ± 18.68 ^b^	132.11 ± 6.35 ^a^
90	Benzeneacetonitrile	-	180.86 ± 0.43 ^a^	155.06 ± 9.97 ^b^	72.15 ± 1.50 ^c^

Note: ‘-’, no odor description information was found in the literature; ‘n.d’, odor thresholds were not found in the ‘Compilations of flavour threshold values in water and other media’ and literature; Different letters in the same row indicated that the contents of aroma compounds had significant differences (*p* < 0.05).

## Data Availability

The data presented in this study are available on request from the corresponding author.

## Supplementary Materials

The following supporting information can be downloaded at: https://www.mdpi.com/article/10.3390/foods11121694/s1, Table S1. The information on 519 non-volatile metabolites, Table S2. The information on 24 differential metabolites, Table S3. The relative content of 90 volatile components, Table S4. The volatile components with OAVs ≥ 1, Table S5. The volatile components of OAVs ≥1 and VIP > 1, Figure S1. The schematic map of sample collection.
