# Supplementary figures and images for: Non-Targeted Metabolomics Analysis Revealed the Characteristic Non-Volatile and Volatile Metabolites in the Rougui Wuyi Rock Tea (Camellia sinensis) from Different Culturing Regions
Source: Foods. 2022 Jun 9;11(12):1694. doi: 10.3390/foods11121694 (PMC9222269; doi:10.3390/foods11121694)

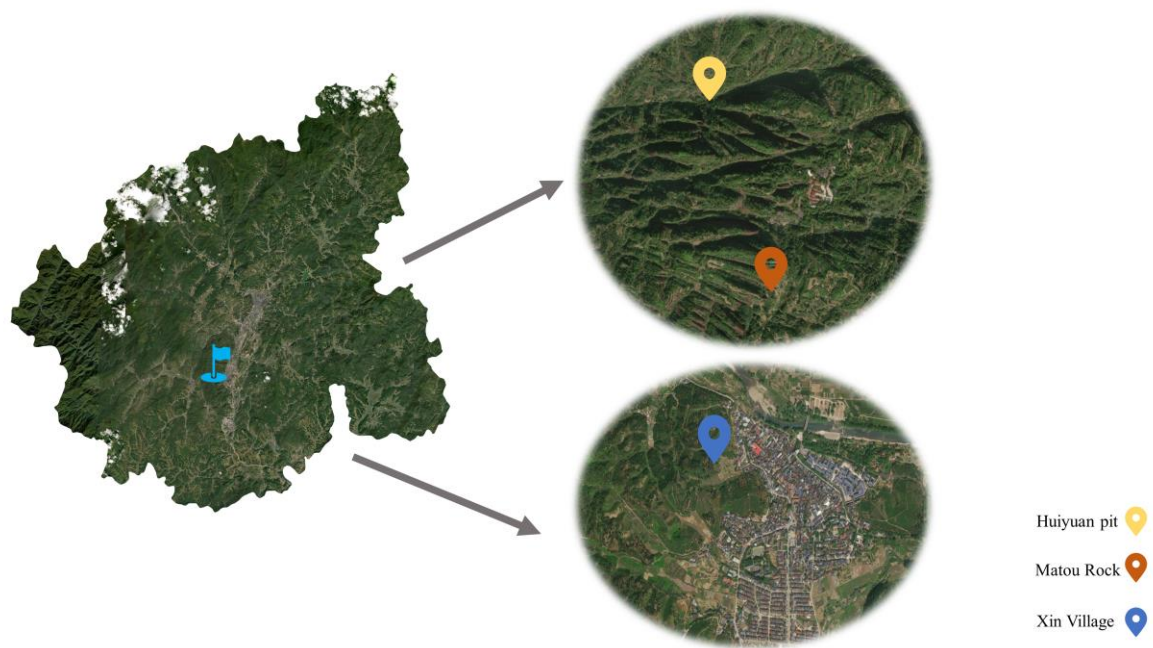

**Figure S1.** The schematic map of sample collection.

Supplement: Supplementary file 1 [file foods-11-01694-s001.zip › Figure S1.pdf]
